# Improvement in the prediction power of an astrocyte genome-scale metabolic model using multi-omic data

**DOI:** 10.3389/fsysb.2024.1500710

**Published:** 2025-01-03

**Authors:** Andrea Angarita-Rodríguez, Nicolás Mendoza-Mejía, Janneth González, Jason Papin, Andrés Felipe Aristizábal, Andrés Pinzón

**Affiliations:** ^1^ Departamento de Nutrición y Bioquímica, Facultad de Ciencias, Pontificia Universidad Javeriana, Bogotá, Colombia; ^2^ Laboratorio de Bioinformática y Biología de Sistemas, Universidad Nacional de Colombia Bogotá, Bogotá, Colombia; ^3^ Department of Biomedical Engineering, University of Virginia, Charlottesville, VA, United States; ^4^ Department of Medicine, Division of Infectious Diseases and International Health, University of Virginia, Charlottesville, VA, United States; ^5^ Department of Biochemistry and Molecular Genetics, University of Virginia, Charlottesville, VA, United States

**Keywords:** genome-scale metabolic models, transcriptome, proteome, dimensional reduction, astrocyte

## Abstract

**Introduction:**

The availability of large-scale multi-omic data has revolution-ized the study of cellular machinery, enabling a systematic understanding of biological processes. However, the integration of these datasets into Genome-Scale Models of Metabolism (GEMs) re-mains underexplored. Existing methods often link transcriptome and proteome data independently to reaction boundaries, providing models with estimated maximum reaction rates based on individual datasets. This independent approach, however, introduces uncertainties and inaccuracies.

**Methods:**

To address these challenges, we applied a principal component analysis (PCA)-based approach to integrate transcriptome and proteome data. This method facilitates the reconstruction of context-specific models grounded in multi-omics data, enhancing their biological relevance and predictive capacity.

**Results:**

Using this approach, we successfully reconstructed an astrocyte GEM with improved prediction capabilities compared to state-of-the-art models available in the literature.

**Discussion:**

These advancements underscore the potential of multi-omic inte-gration to refine metabolic modeling and its critical role in studying neurodegeneration and developing effective therapies.

## 1 Introduction

Astrocytes perform essential functions in the central nervous system (CNS) for the maintenance of its function and health. The inflammatory response of these cells can be triggered as part of a process termed astrogliosis, which has been widely associated with neurodegeneration (Osorio et al., 2020; Phatnani and Maniatis, 2015; Takuma et al., 2004; Sofroniew and Vinters, 2010). Astrogliosis is a cell response to several challenges in the CNS, such as injuries, infections, or diseases, which involve molecular, morphological, and functional changes (Escartin et al., 2019). However, the precise mechanism by which this response becomes neurodgenerative is yet to be described. To that end, genome-scale metabolic models (GEMs) have been crucial to better understanding underlying relationships with neurodegenerative diseases (Nielsen, 2017). For example, previous efforts in our laboratory had led to the identification of several metabolic pathways involved in palmitic acid (PA)-induced toxicity using hybrid computational-wet lab strategies (Osorio et al., 2020; Angarita-Rodríguez et al., 2022; Vesga‐Jiménez et al., 2022).

**FIGURE 1:**
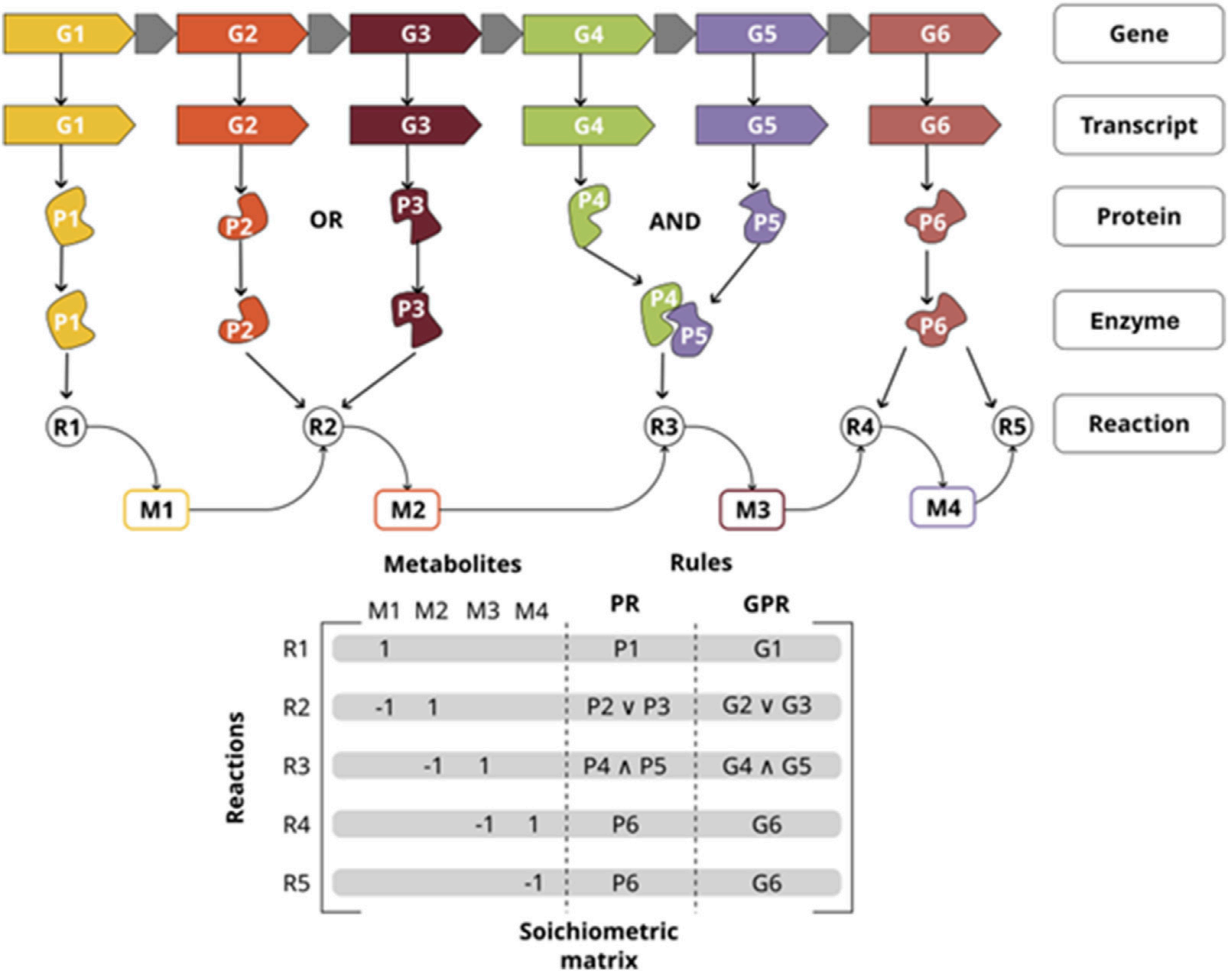
Gene-Protein-Reaction (GPR) rules map the relationship between key components of a metabolic network reconstruction. Those interactions are represented as GPR rules in the GEM (OR, AND); and how they are also associated with the stoichiometric matrix. P1 is the only gene necessary for reaction R1 to take place. either P2 or P3 are necessary so reaction R2 takes place. Genes P4 and P5 must be expressed so reaction R3 can take place.

GEMs are a highly used approach in systems biology with applications ranging from the basic understanding of genotype-phenotype relationships to solutions in industrial biotechnology and systems medicine (Bernstein et al., 2021; Wang et al., 2021a). GEMs are mathematical representations of the metabolic network of an organism at a system level, representing its entire metabolic functions, mainly through the definition of a stoichiometric matrix, which represents the relations between reactions and metabolites (Orth et al., 2010). Another essential aspect of GEMs is the definition of gene-protein-reaction (GPR) rules that associate the biochemical reactions to their corresponding genes and enzymes (Figure 1). The latter is used to contextualize species-specific knowledge and omic data from different sources, by mapping them into the metabolic network. In this sense, many diseases that are attributed to metabolic disorders, including cancer and neurodegenerative diseases, have been modeled through this approach (Osorio et al., 2020; Somvanshi and Venkatesh, 2014; Wang et al., 2021b); it is important to highlight that GEMs have also been used to describe the metabolic condition of specific tissues, cell types, and contexts at the system level by integrating omic data which better reflects the state of the cell (Bernstein et al., 2021; Wang et al., 2021b; Passi et al., 2022).

In recent years, high-throughput techniques have gained popularity for the study of neurodegenerative diseases, mainly for the identification of relationships between the measured molecules and neuronal degeneration (Diaz-Ortiz and Chen-Plotkin, 2020). Even though the omic data produced by these techniques provide a holistic view of the organism, the analysis of data from a single omic source is a challenge itself due to its complexity and size, as well as the fact that a single omic source only represents one step of a series of complex steps in cellular behavior, something that prevents us from predicting more complex cellular phenotypes. Therefore, since most biological mechanisms involve multiple biomolecules, a single omic source misses the interaction between these biological layers (Hassan et al., 2022; Haas et al., 2017). In this sense, GEMs work as a framework to integrate and analyze multi-omic data while considering these interactions, allowing us to give plausible predictions closer to biological reality (Kim et al., 2016; Cho et al., 2019; Väremo et al., 2015).

To date, all genome-scale metabolic models of astrocytes (Osorio et al., 2020; Martín-Jiménez et al., 2017) are reconstructed with transcriptome data and information gathered from the literature; but they are not contextualized with multi-omic data. One of the state-of-the-art GEMs for astrocytes was created by our laboratory using multi-omic data (Osorio et al., 2020). Following these efforts, in the current work, we reconstructed a tissue-specific human astrocyte GEM based on transcriptomic and proteomic data directly obtained by our laboratory. The experimental data were taken from astrocytes under basal conditions, stimulated with PA, and pre-treated with tibolone (Tb) followed by PA stimulus, the same biological scenarios used by (Osorio et al., 2020).

However, although rich in biological information, both transcriptome and proteome data have shortcomings when used independently to reconstruct a GEM. Specifically, it is hard to extrapolate the metabolic fluxes from the gene expression levels observed in transcriptomic data (Cho et al., 2019). In contrast, the proteome is closer to the metabolic activity, but its coverage and accuracy are often lower than the former data type (Cho et al., 2019). To address these limitations, several studies have proposed methods that integrate transcriptomic and proteomic data. For instance (Bordbar et al., 2014), introduced GIMMEp to overcome the limitations of algorithms that rely on predefined objective functions to create context-specific GEMs. This algorithm involves three steps: defining objective functions from proteome data, extracting individual subnetworks that meet each objective function based on transcriptomic data, and combining these subnetworks to form the final GEM. However, although this approach provides a robust framework, it can expand the solution space when applied to astrocyte GEMs with well-defined objective functions, potentially making the resulting model less predictive than desired.

In another interesting approach (Väremo et al., 2015), the proteome is assessed, contrasting highly abundant proteins with the transcriptome; then, the genes with high expression are added to the subset of abundant proteins supported by the transcriptome. In this approach, the transcriptome is considered the ground truth, ignoring its limitations and using it to evaluate proteome data, without considering the reverse evaluation.Haga clic o pulse aquí para escribir texto.

In our approach, we propose a novel integration of transcriptomic and proteomic data by leveraging the gene-protein-reaction rules included in GEMs. Instead of assessing each data type separately, we perform dimensional reduction on the combined data using principal component analysis (PCA), creating a single-vector representation. To our knowledge, this approach has been applied to dimensionally reduce RNAseq data but not protein abundance data (Ou et al., 2021). We then used this single-vector to contextualize a human GEM model, leading to the most comprehensive and accurate astrocyte-specific GEM to date.

## 2 Materials and methods

### 2.1 Transcriptomic and proteomic data

The proteome and transcriptome data were derived from a human astrocyte cell line (Lonza), cultured in Astrocyte Basal Medium supplemented with SingleQuots (Lonza). They were cultured at 37 °C and 5% of CO2. Then, the respective treatments were applied to obtain data for the following three scenarios: astrocytes under basal conditions, stimulated with Palmitic Acid (PA), and pre-treated with Tibolone (Tb) followed by PA stimulus. The Tb pre-treatment at 10 mM was applied for 24 h (Martin-Jiménez et al., 2020); then the cells were treated with concentrations 2000 µM of PA during 24 h (Martin-Jiménez et al., 2020); finally, the control group (under basal conditions) included bovine serum albumin, free of fatty acids, and carnitine at 2 mM (Martin-jiménez et al., 2020).

To profile the transcriptome, the total RNA was extracted with the RNeasy mini kit (Qiagen, United States), following the manufacturer’s instructions. RNase-free DNase I was used to avoid contamination with genomic DNA. Samples were stored at −80°C in a nuclease-free buffer to be sequenced on an Illumina HiSeq 2000 machine with a 150 bp paired-end configuration, yielding ∼75 million reads per sample. This data is available in the Gene Expression Omnibus or GEO repository under the identifier GSE166500 (https://www.ncbi.nlm.nih.gov/geo/query/acc.cgi?acc=GSE166500). We assessed RNA-seq quality with QUARS (QUAlity control for RNA-Seq; github.com/tluquez/QUARS), a workflow that integrates several tools included in Nextflow (v18.10.1). Following best practices (Conesa et al., 2016), the genes that had fewer than 10 total reads were discarded after adding the read counts from technical and biological replicates on any treatment.

For protein extraction, the medium was removed, and the container was cleaned with 1 mL of cold 1X PBS, then extracted with a pipette. Then, a preparation of 72 mL of the RIPA cocktail plus protease inhibitors were added. The samples were then centrifuged at 15,200 rpm and −4°C for 13 min. The proteins were quantified by the bicinchoninic acid method (BCA1 Sigma-Aldrich kit). The digested peptides were analyzed by LC-MS/MS in a Thermo Scientific Q-Exactive Orbitrap mass spectrometer together with Proxeon Easy-nLC II HPLC (Thermo Scientific) and a Proxeon nanospray source. The MS/MS spectrum was obtained using a method of the top 15, where the 15 main ions of the spectrum were subjected to a HCD (High Energy Collisional Dissociation). In selecting the precursor ions, an isolation mass window of 2.0 m/z was used, and fragmentation was done at 27% normalized collision energy. For dynamic exclusion, a duration of 22 s was used.

In the processing of the resulting files, the following parameters were used: maximum 2 missing cleavages, minimum 50% in the identification of ion precursors, and search for razer and unique peptides. Valid proteins were obtained from the SwissProt human database. Using the program proteome discoverer 2.3, they are quantified without labels in the search engine Sequest and AMANDA. In addition, MaxQuant v1.6.10.43 v1.6.10.45 and Perseus were used to identify valid proteins.

Finally, to obtain the relative count, non-normalized protein intensities were imported into R version 4.0.1 (R Core Team, 2019). First, the data were transformed with log2 to obtain a more symmetric distribution before analysis, and all proteins were maintained with 70% of valid values per group, that is, 6 replicates per group (Karpievitch et al., 2012); In the transcriptomic data analysis, the raw counts were transformed into log2 scale to achieve a more symmetric distribution, which facilitates comparison between groups and reduces skewness in the data. This transformation, along with VSN variance stabilization, ensured that the variance remained constant across the data range, thus enhancing the interpretability of the results. As with protein data, missing values in the transcriptomic dataset were imputed using K-Nearest Neighbor (KNN) Imputation, with a KNN value of 10, following recommended best practices (Chai et al., 2014; Välikangas et al., 2017). For this a KNN value of 10 was used as suggested by (Välikangas et al., 2017).

### 2.2 Data mapping to reactions

**FIGURE 2:**
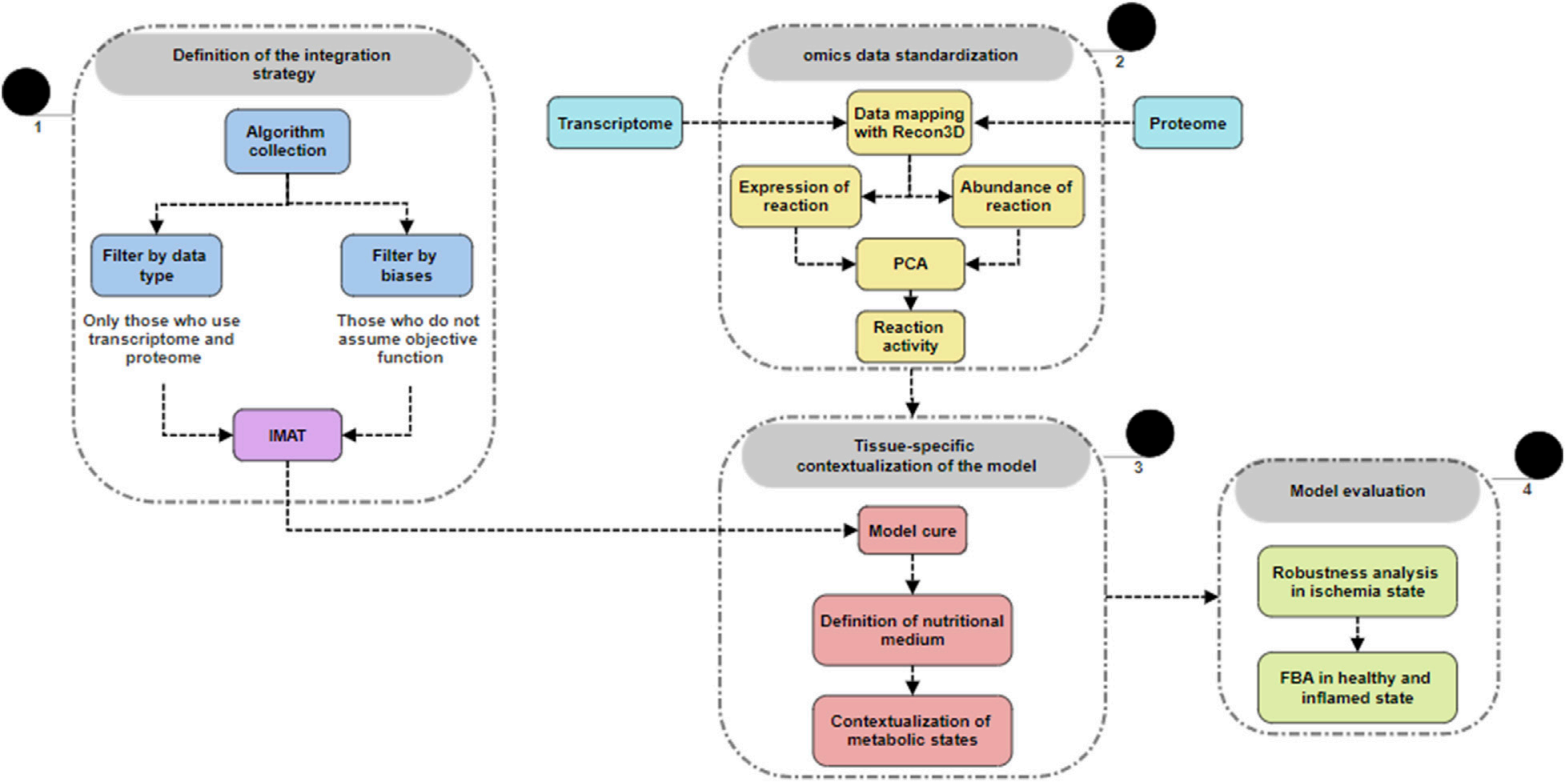
General overview of methodology. 1) Overview of the methodology used for integrating transcriptomic and proteomic data, highlighting key steps and outcomes. 2) Raw data obtained and deposited by our laboratory (GSE166500) were retrieved and mapped to the generic Recon3D model. The dimensionality of the transcriptomic and proteomic data was reduced, and then, 3) these data were used to restrict the generic model, generating a context-specific model. The model was further curated using the *fastGapFill* algorithm, with restrictions defined according to the metabolic state context. 4) The restricted model was subjected to flux balance analysis and other optimization-based algorithms.

We next map RNAseq and protein abundance data to each reaction present in the model. To do so, we use Recon3D’s GPR association rules, which use Boolean expressions to encode non-linear relationships between genes, proteins, and reactions (Di Filippo et al., 2024) (Figure 2, part 2). In this sense, when there is more than one entity related to a single reaction, we take the minimum value when the relationship between the entities is AND, and the maximum when the relationship is OR (Figure 2). As a result, we obtain two ℝn vectors for each dataset, where n is the number of reactions. One vector represents the abundance of the enzymes that catalyze the reaction, based on the proteome data, while the other vector represents the expression of the genes that code for those enzymes, based on the transcriptome data.

### 2.3 Principal component analysis: transformation of transcriptome and proteome data

A methodology to transform transcriptome and proteome data has been previously described and published by our research group (Angarita-Rodríguez et al., 2022). This method generates a single vector that integrates the information from both measurements, addressing the lack of expected linear correlation between protein abundance and gene expression (Figure 2, part 2).

Before performing the principal components analysis (PCA), we standardized (z-score scaling) the input data. Because the scale difference between the transcriptome and proteome can distort the PCA (Jollife and Cadima, 2016). The standardization, also known as z-score scaling, was performed by subtracting the mean from each value and dividing by the standard deviation. Subsequently, the variance and covariance matrix were calculated, obtaining the eigenvalues and eigenvectors, as well as the principal components. This process was implemented using MATLAB^®^ (Hyduke et al., 2011).

### 2.4 Context-specific model reconstruction

We employed iMAT to contextualize the model (Zur et al., 2010). This method uses two manually selected thresholds to categorize expression and abundance levels into three states: high, medium and low. These states help identify active reactions that should be included in the GEM (Figure 2, part 1). Due to distribution skewness, we applied a logaritmic transformation, which stabilizes variance and improves the reliability of the thresholds iMAT uses to classify reactions by metabolic activity levels. Afterward, we used the single-dimensional vector obtained from PCA as described above to reduce the base model, Recon3D (Brunk et al., 2018), by applying the iMAT implementation from the COBRA toolbox v3.0 (Heirendt et al., 2011) through the function *createTissueSpecificModel*. This algorithm identifies a balance between the inclusion of reactions with high activity and the elimination of the low ones (Zur et al., 2010). The defined thresholds to classify the reactions by activity were −500 for the low threshold, and 500 for the high one. Lastly, we used the *exp2flux* algorithm (Osorio et al., 2016) to create three astrocyte models: healthy, inflamed, and tibolone pre-treatment. As the input, we used dimensionally-reduced data from each specific scenario as described in the previous section.

### 2.5 Model curation

#### 2.5.1 Gap-filling

The reconstruction process often leaves some gaps in the model, which are identified from metabolites that can be produced but not consumed, or *vice versa*. We used the *fastGapFill* algorithm (Thiele et al., 2014) in the COBRA Toolbox v3.0 (Heirendt et al., 2011), which uses the universal biochemical reaction databases to fill the gaps with stoichiometrically consistent reactions (Figure 2, part 3).

#### 2.5.2 Stoichiometric consistency

To ensure the consistency of the model, we searched for leak and siphon metabolites with the method *findMassLeaksAndSiphons* available in the COBRA Toolbox v3.0 (Heirendt et al., 2011). Leak metabolites are those that are still produced when closing the nutritional inputs of the model. Therefore, they are produced “from nothing”. Siphon metabolites are those produced when closing the outputs; thus, they are consumed “for nothing”. The presence of these species in the model implies a violation of the law of mass conservation (Heirendt et al., 2011). In this sense, we identified the minimum set of reactions responsible for each leaked and siphoned metabolite by using the function *findMinimalLeakageModeRxn* available in the COBRA Toolbox v3.0 (Heirendt et al., 2011). Then, we identified pairs of reactions with common metabolites, one on the product side and the other on the reactant side. Lastly, we used the Stoichiometric tools package to search for the null space in the elements matrix for each pair of reactions, and the missing metabolites to balance the reaction were added.

#### 2.5.3 Nutritional input definition

As the composition of ABM (Advanced Basal Medium) is not available, we defined the nutritional input based on the composition of Dulbecco’s Modified Eagle Medium (DMEM) enriched with fetal bovine serum (FBS) at 10%, which is the same one used by Osorio et al. (2020). Then, we took the metabolic composition of DMEM defined by Tejera et al. (2020). Conversely, as FBS is a natural medium, the metabolite composition and concentration can vary significantly, even between batches from the same supplier, which can introduce variability in experimental outcomes and affect the consistency of metabolic analyses (Yang and Xiong, 2012). Thus, we used different sources to build Supplementary Table 2 (Yang and Xiong, 2012; Branzoi et al., 2010).

### 2.6 Simulation and prediction assessment

To simulate the experimental conditions, we converted the millimolar (mM) concentrations of the relevant metabolites into metabolic fluxes (mmol gDW⁻^1^ h⁻^1^) using a standard method. Specifically, the concentrations were normalized based on cell dry weight (gDW), assuming a standard composition of the astrocyte cells. For each scenario, the substrate concentrations (e.g., palmitic acid, Tibolone) were converted into corresponding flux values using the method described by (Shinfuku et al., 2009), where metabolite uptake or secretion rates were calculated based on the experimental concentration values. This approach allowed us to integrate experimentally derived concentrations into the Flux Balance Analysis (FBA) framework for accurate simulation of metabolic activities under each condition.

Therefore, we defined four metabolic scenarios: (i) a basal state, simulating the standard conditions of an astrocyte; (ii) an ischemic condition, in which oxygen and glucose availability were progressively reduced from 2.5 to 0 mmol gDW⁻^1^ h⁻^1^ (millimoles per Gram of dry weight per hour); (iii) a challenge condition with 0.208 mM/g of PA (palmitic acid); and (iv) a pre-treatment with 70 μM/g of Tb (tibolone) followed by exposure to the same concentration of PA (González-giraldo et al., 2019). For each scenario, flux balance analysis (FBA) was performed using the COBRA Toolbox with the loopless flag enabled, minimizing thermodynamically infeasible loops that could otherwise affect predictions (Schellenberger et al., 2011).

In each of these simulations, the same objective function (biomass) was used, but input fluxes were adjusted according to the specific scenario requirements. For instance, in the ischemic condition, the input fluxes of oxygen and glucose were progressively decreased, whereas in the challenge and pre-treatment scenarios, specific fluxes for PA and Tb were added sequentially. This approach allowed us to capture the metabolic adaptations of astrocytes under distinct environmental stresses, providing insights into how these cells manage resources in different contexts.

Finally, the models by Osorio et al. (2020) and our model were evaluated against experimental flux data obtained from Amaral et al. (2011). After incorporating the nutritional input, a flux balance analysis (FBA) was performed to calculate the biomass value, which was then compared with experimentally measured values reported by Amaral et al. (2011). Subsequently, the objective function was fixed at 0.32, and a flux variability analysis (FVA) was conducted. This analysis allowed for a comparison of the flux ranges of key reactions with experimental metabolite values.

## 3 Results

### 3.1 Integration of omic data

**FIGURE 3:**
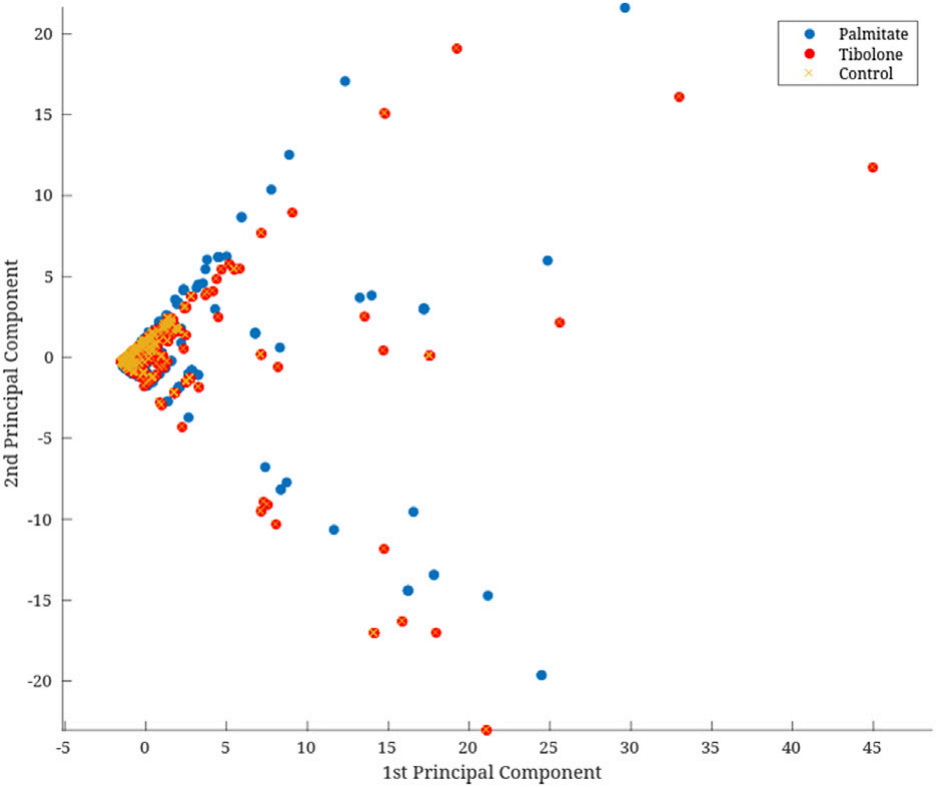
Dimensional reduction with PCA of omic data. This PCA plot represents the multi-omics data (e.g., transcriptomic and proteomic profiles) across various experimental conditions or biological groups. Each point corresponds to a sample, color-coded by condition, highlighting the variance in omics profiles between groups. The axes represent the first two principal components (*x*-axis as PC1 (60.8% variation); *y*-axis as PC2 (31.0% variation), which capture the highest variance in the dataset.

In the Recon3D model, only 56.7% of the reactions are associated with one or more genes through the GPR rules, and only 49.9% of the reactions are linked to one or more proteins. This means that experimental data can contextualize only about half of the metabolic network in the model. After mapping the experimental data to the GPRs, we performed a Principal Component Analysis (PCA) on the mapped data. As a complementary step, individual PCAs were performed for gene expression and protein abundance data, and these results are shown in Supplementary Figure 1. Based on the screen plot (Figure 3) and the proportion of variance explained criterion, we selected the first two principal components for dimensionality reduction, as described in the methodology. The resulting vector was correlated with each environment to assess how well it represented the original data. Therefore, we applied a logarithmic transformation to the resulting vector to improve iMAT performance, which relies on a threshold-based approach to identify the reactions that should be retained in the model.

### 3.2 Tissue-specific astrocyte GEM

Given that only approximately 50% of the reactions were mapped using Recon3D, we can hypothesize that the other 50% of reactions present in the RNAseq/proteome dataset may not have been fully captured. This suggests that there could be additional reactions in the dataset that are either context-specific or not represented in the base model. As a result of the contextualization process, we obtained a tissue-specific GEM that includes 6,520 metabolites and 10,586 reactions, with 5,297 reactions being reversible and 5,289 irreversible. The integration of omics data likely contributed to the unique reactions captured in this model, reflecting the metabolic activities specific to the tissue or condition being studied.

**FIGURE 4:**
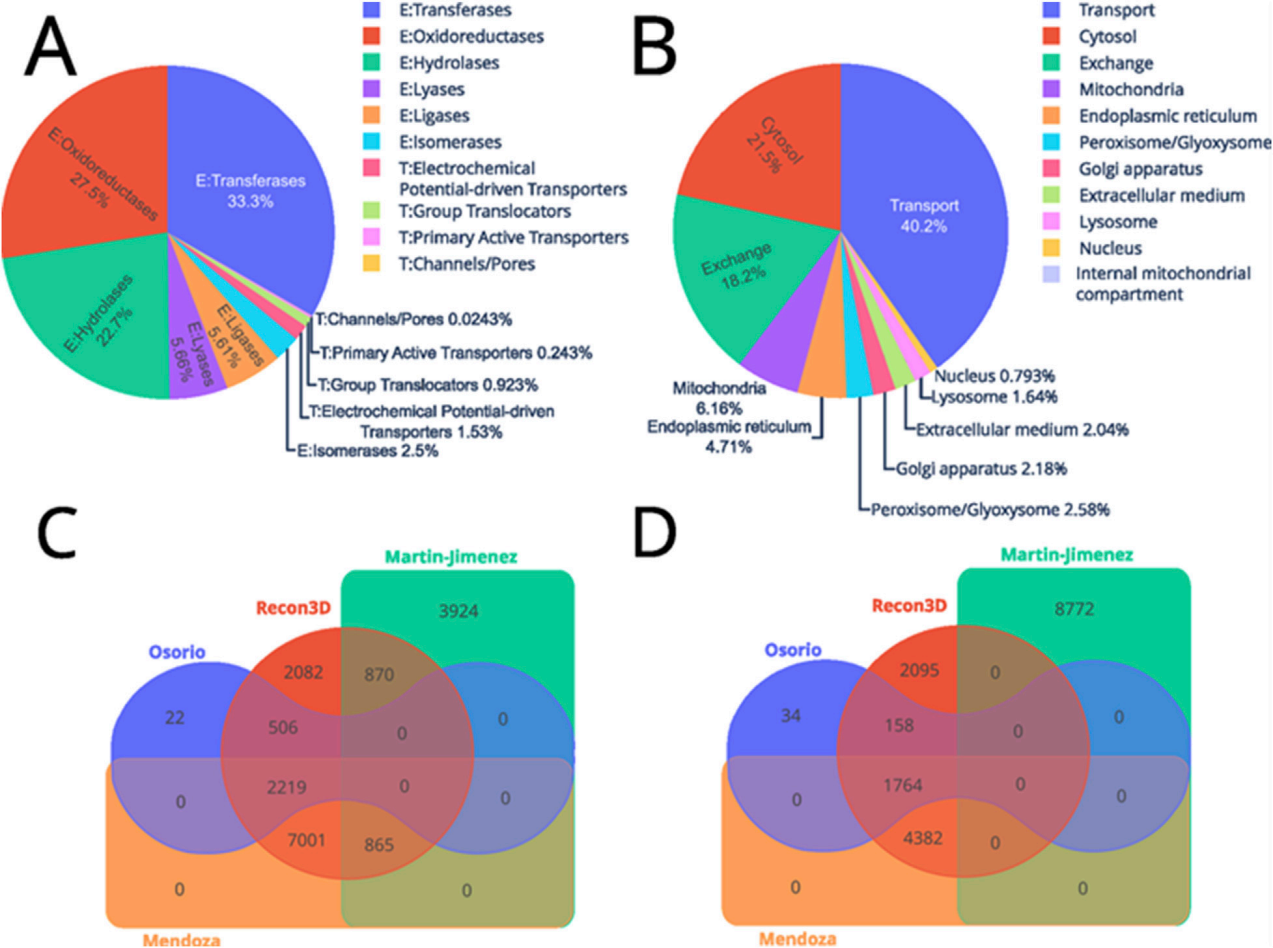
Distribution of the enzymes and reactions included in the astrocyte GEM. The upper part depicts the reactions classified by **(A)** type of protein, where those that begin with “E:” are enzymes and “T:” are transport proteins. While **(B)** shows the distribution by cellular compartment. Conversely, the lower part shows two Venn diagrams of the similarities of metabolites **(C)** and reactions **(D)** between the different models.

Thus, this model accounts for 9 compartments that include cytosol [c], extracellular medium [e], Golgi apparatus [g], internal mitochondrial compartment [i], lysosome [l], mitochondria [m], nucleus [n], endoplasmic reticulum [r], and peroxisome/glyoxysome [x]. Figure 4A shows the distribution of enzymatic functions based on Enzyme Commission (EC) numbers and identifiers from the Transporter Classification Database (TCDB). Regarding the exact distribution of the enzymatic function: 63 electrochemical potential-driven transporters, 38 group translocators, 10 primary active transporters, and 1 channel/pore. In contrast, 1,371 enzymes are transferases, 1,132 oxidoreductases, 933 hydrolases, 233 lyases, 231 ligases, and 103 isomerases. Nevertheless, out of the 5,880 reactions that are not in compartments: 1,834 exchanges and 4,056 are transport reactions. While the compartments are divided as follows: 2,167 were in the cytosol, 621 in the mitochondria, 475 in the endoplasmic reticulum, 269 in the peroxisome/glyoxysome, 220 in the Golgi apparatus, 206 in the extracellular medium, 165 in the lysosome, and 80 in the nucleus (Figure 4B).

To assess the completeness of our multi-omics model, we compared it against those from (Osorio et al., 2020) and (Martín-Jiménez et al., 2017), we examined the reaction and metabolite identifiers across these models, Recon 3D, and the model reconstructed here (Figures 4C, D). For clarity, we will refer to the models by the last name of the associated authors.

The comparison of reaction identifiers revealed that the Osorio (blue) and Martín-Jiménez (green) models do not share common reactions due to differences in identifier systems, indicating potential variations in how reactions are represented or defined. In contrast, the Recon3D model (red) shares reactions with all other models, suggesting it provides broader coverage of common metabolic reactions. The Mendoza model (orange) includes an additional 7,001 reactions that are not found in existing models, suggesting a significant expansion or enhancement in the metabolic representation. The inclusion of these additional reactions could be due to several factors. First, the Mendoza model might have integrated reactions from other data sources or experimental evidence not covered by Recon3D, such as organism-specific pathways or context-dependent reactions. Additionally, this expansion may result from manual curation or the integration of omics data (e.g., transcriptomics and proteomics) that suggest context-specific metabolic activities.

Moreover, Mendoza shares 2,219 reactions with Osorio and 865 with Martín-Jiménez. This implies that some reactions shared between Martín-Jiménez and Recon3D were not active in the astrocyte model, and further, 506 reactions shared by Osorio and Recon3D were omitted from Mendoza based on experimental data.

Regarding metabolites, Martín-Jiménez does not share any metabolites with other models, due to a different notation system for metabolite identification. Nevertheless, Recon3D and Osorio each have unique metabolites—2,095 in Recon3D and 34 in Osorio. They also share 158 metabolites not found in the other models. Mendoza shares 4,382 metabolites solely with Recon3D and an additional 1,764 with both Recon3D and Osorio. This comparison highlights the quality and completeness of the reconstructed model relative to existing ones. A model that shares many reactions and metabolites with others is generally more consistent with prior knowledge, whereas models introducing new reactions may offer novel insights. Mendoza’s inclusion of additional reactions and metabolites suggests improvements in metabolic representation, potentially enhancing the accuracy of metabolic simulations.

During the model curation, we identified 2,205 leaks and 2,312 siphon metabolites. Thus, as described in the methods, we stoichiometrically balanced each reaction and pairs of inconsistent reactions. As a result, we eliminated the leak metabolites, but none of the siphon metabolites were removed. Therefore, during the simulations, we optimized the model with loopless COBRA, to discard the thermodynamically infeasible cycles that may then be generating erroneous outcomes (Schellenberger et al., 2011). We applied the nutritional input composition to the boundaries of the model.

### 3.3 Model predictions and experimental comparisons

Using Flux Balance Analysis (FBA) as described above, our model predicted a biomass flux of 0.33 mM gDW⁻^1^ h⁻^1^ (Table 1), employing the biomass function defined in Recon3D. In the case of astrocytes, which do not primarily grow in terms of biomass, the predicted biomass flux in the model could instead be interpreted as a measure of cellular maintenance rather than actual proliferation (Verkhratsky and Nedergaard, 2018; Çakir et al., 2007). Astrocytes engage in metabolic activities that maintain their cellular function and homeostasis rather than supporting rapid biomass accumulation (Çakir et al., 2007). From the model’s perspective, biomass synthesis predictions are being calculated, but in reality, the experimental data (based on transcriptomic and proteomic measurements) likely reflect the metabolic activity required for maintaining the cell’s integrity and functionality rather than growth *per se*.

**TABLE 1:** Comparison of metabolic fluxes for different models without fixing the biomass objective function against experimental data and reported experimental values by Amaral et al. (2011) (mmol gDW⁻^1^ h⁻^1^).

Model	Biomass	Glutathione	Glutamine	Lactate
Experimental	0.32	00,015	0.26	0.158
Mendoza Healthy	0.33	0.74	1.25	0.68
Osorio Healthy	0.37	0.3	3.11	1.06
Mendoza PA	0.22	0.204	0.59	0.30
Osorio PA	0.18	0.35	2.13	1.53

The experimental data, which include transcriptomic and proteomic profiles, were derived from cultured human astrocytes (Lonza) under different conditions (basal, stimulated with palmitic acid, and pre-treated with Tibolone followed by PA stimulus). These datasets represent the metabolic shifts occurring under these conditions and provide insights into how astrocytes adjust their metabolic processes to maintain cellular homeostasis rather than focusing on biomass growth. Therefore, using the biomass objective function could serve as a proxy for the overall metabolic load required for maintenance.

**FIGURE 5:**
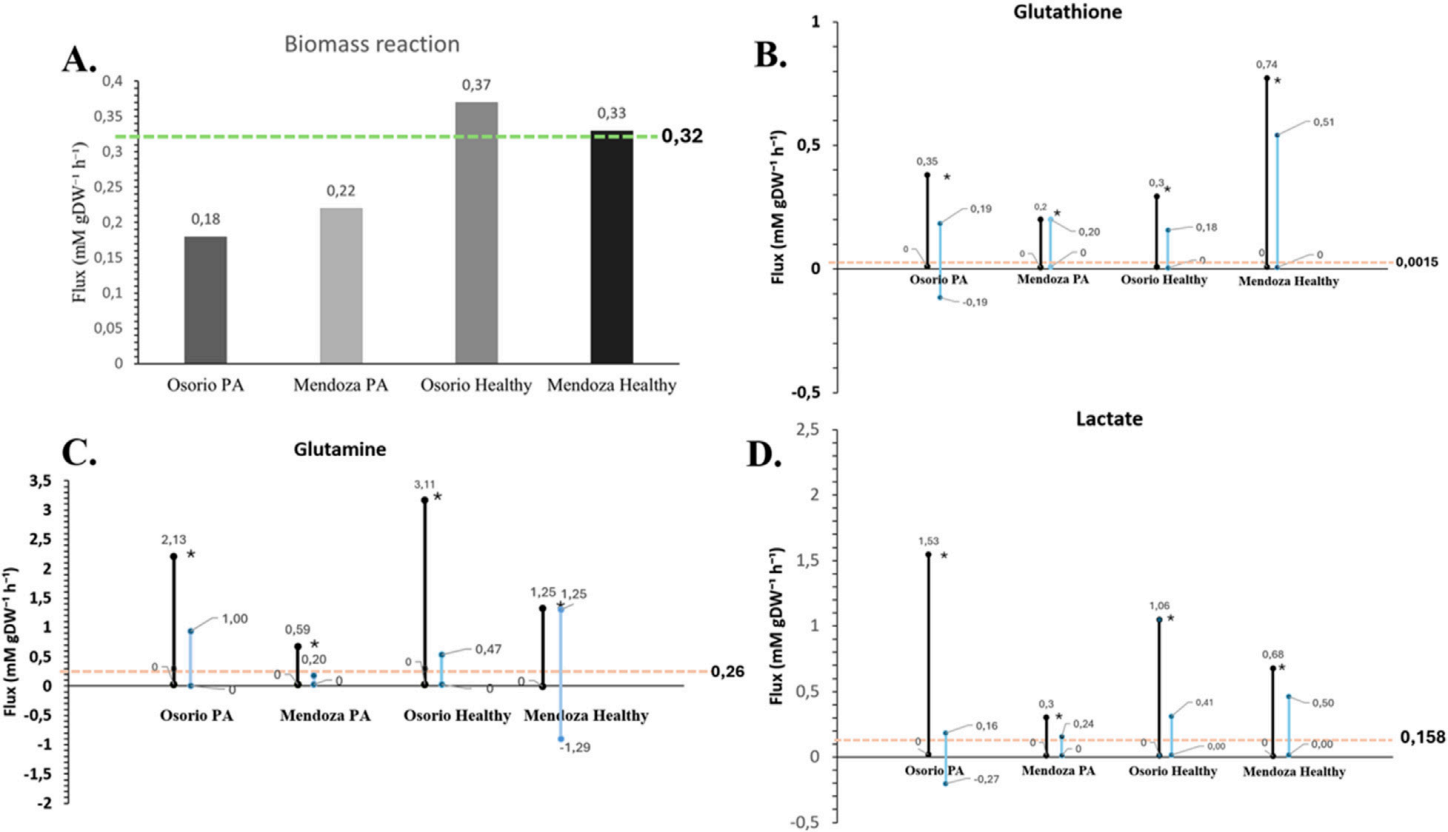
Validation of the astrocyte model against experimental data. The figure illustrates the validation of the astrocyte model by comparing simulated metabolic fluxes with experimental measurements. The pink line represents the experimentally determined flux value for each metabolite (Amaral et al., 2011) (mmol gDW⁻^1^ h⁻^1^). The blue bars depict the flux variability ranges derived from a flux variability analysis (FVA), with the objective function constrained to a value of 0.32, as reported in experimental data (Prah et al., 2019). The black bars indicate the FVA-derived flux ranges without the application of this constraint. Dots represent the optimized flux values obtained through flux balance analysis (FBA) for the corresponding exchange reactions. **(A)** Comparison of biomass values between the models and the reported experimental value. **(B)** Flux Variability Analysis (FVA) for glutathione in each model; the pink line indicates the experimentally measured value. **(C)** Flux Variability Analysis (FVA) for glutamine in each model; the pink line indicates the experimentally measured value. **(D)** Flux Variability Analysis (FVA) for lactate in each model; the pink line indicates the experimentally measured value. The findings indicate a strong concordance between the model-predicted fluxes and experimental data, with the experimentally observed values consistently falling within the simulated flux ranges.

This prediction is in close agreement with the *in vitro* growth flux of 0.32 mM gDW⁻^1^ h⁻^1^ reported by Prah et al. (2019), which is closer to our value than the 0.37 mM gDW⁻^1^ h⁻^1^ reported in the latest model by Osorio et al. (2020) (Figure 5A). It is important to note that the experimental value was converted from cell count to mM gDW⁻^1^ h⁻^1^ using the method proposed by Shinfuku et al. (2009).

### 3.4 Metabolic simulations

The biomass growth predictions from our models were consistent and closely matched the reported experimental value (Prah et al., 2019) (Figure 5). However, for the metabolites analyzed (lactate, glutamine, and glutathione), the flux values predicted by flux balance analysis (FBA) deviated from experimental values. Upon performing a flux variability analysis (FVA) (Table 2), we obtained flux ranges for each metabolite when the objective function was fixed at 0.32 mm gDW⁻^1^ h⁻^1^, consistent with the experimentally reported biomass value (Figure 5A).

**TABLE 2:** FVA Flux values for each reaction with the objective function fixed at 0.32 mmol gDW⁻^1^ h⁻^1^, corresponding to the experimentally measured value in the healthy state (mmol gDW⁻^1^ h⁻^1^) (Amaral et al., 2011).

Reaction model	Min	Max	Expermental value
Glutamine _ Mendoza_Healthy	−1.29	1.25	0.26
Glutamine _ Osorio_Healthy	0	0.47	0.26
Gluthation_Mendoza_Healthy	0.00	0.51	00,015
Gluthation_Osorio_Healthy	0.00	0.18	00,015
Lactate _Mendoza_Healthy	0.00	0.50	0.158
Lactate _Osorio_Healthy	0.00	0.41	0.158
Glutamine _ Mendoza_PA	0.00	0.20	NA^a^
Glutamine _ Osorio_PA	0.00	1.00	NA^a^
Gluthation_Mendoza_PA	0.00	0.2	NA^a^
Gluthation_Osorio_PA	−0.19	0.19	NA^a^
Lactate _Mendoza_PA	0.00	0.24	NA^a^
Lactate _Osorio_PA	−0.27	0.16	NA^a^

^a^ At this moment we do not have experimental value for the metabolites associated with the inflammatory state.

The flux results indicate that the experimental values fall within the predicted ranges, and by fixing the experimental biomass growth value, the metabolite flux ranges become even closer to the experimental values (Figures 5B–D). For example, (Amaral et al., 2011), reported a glutamine flux of 0.26 mM gDW⁻^1^ h⁻^1^, while the (Osorio et al., 2020) model predicted a flux of 3.11 mM gDW⁻^1^ h⁻^1^ for control astrocytes and 2.13 mM gDW⁻^1^ h⁻^1^ for PA-treated astrocytes. Our model predicts a flux of 1.25 mM gDW⁻^1^ h⁻^1^ for control astrocytes and 0.47 mM gDW⁻^1^ h⁻^1^ for PA-treated astrocytes (Figure 5C).

Notably, the experimental flux data used in our comparison do not incorporate certain metabolic pathways that could significantly contribute to the patterns observed in our model. The metabolic flux data comprise 47 fluxes, 10 of which were measured experimentally, either as production or consumption rates of glucose, lactate, alanine, glutamine, leucine, isoleucine, valine, and cystine, or obtained from the literature (citrate and glutathione release rates) (Amaral et al., 2011). These fluxes were derived through metabolic flux analysis (MFA), which calculates fluxes based on experimental measurements rather than flux balance analysis (FBA). From these data, we infer that astrocytes consistently release glutamine. However, discrepancies between observed fluxes and expected values may be attributed to variations in metabolic activity, substrate availability, or glutamine transport efficiency (Bröer and Brookes, 2001).

Therefore, additional experimental data would be required to reliably identify unknown metabolic fluxes that could influence the optimal values of these metabolic fluxes in the model.

Regarding glutathione, our model predicted metabolic fluxes that differed from the experimental value reported (0.0015 mM gDW⁻^1^ h⁻^1^) (Amaral et al., 2011), although the results of the FVA analysis remain in the lower part of the flux range (Figure 5B). A significant increase in the metabolic flux of a metabolite could reflect changes in the cellular metabolic state, such as stress responses or metabolic alterations, which could impact critical cellular functions, neuronal communication, or the maintenance of the brain’s extracellular environment (Vicente-Gutierrez et al., 2019). For astrocytes, this could affect neurotransmitter regulation, potassium homeostasis, or responses to injury or disease.

For lactate (Osorio et al., 2020), reported fluxes of 0.3 mM gDW⁻^1^ h⁻^1^ for control astrocytes and 1.53 mM gDW⁻^1^ h⁻^1^ for PA-treated astrocytes. Our simulated values of 0.68 mM gDW⁻^1^ h⁻^1^ and 1.06 mM gDW⁻^1^ h⁻^1^ for the control and treated states, respectively, compared to reported values of 0.158 mM gDW⁻^1^ h⁻^1^ under homeostatic conditions, suggest that our predictions are closer to experimental data (Figure 5D). This result further supports the effectiveness of the multi-omics approach utilized in our study.

It is worth noting that out of the 47 fluxes in the metabolic network, 10 were measured experimentally using isotopic transient 13C metabolic flux analysis (MFA) to estimate intracellular fluxes in primary cultures of astrocytes (Amaral et al., 2011). Our predictions indicate a significant glycolytic flux in astrocytes, with approximately 24.2% of the glucose (3.26 mM gDW⁻^1^ h⁻^1^) being converted into lactate (0.79 mM gDW⁻^1^ h⁻^1^). Previously (Ben-Yoseph et al., 1996), reported a basal pentose phosphate pathway flux in astrocytes of approximately 7% of the total lactate produced from glucose, which increased to 67% under oxidative stress conditions.

## 4 Discussion

**FIGURE 6:**
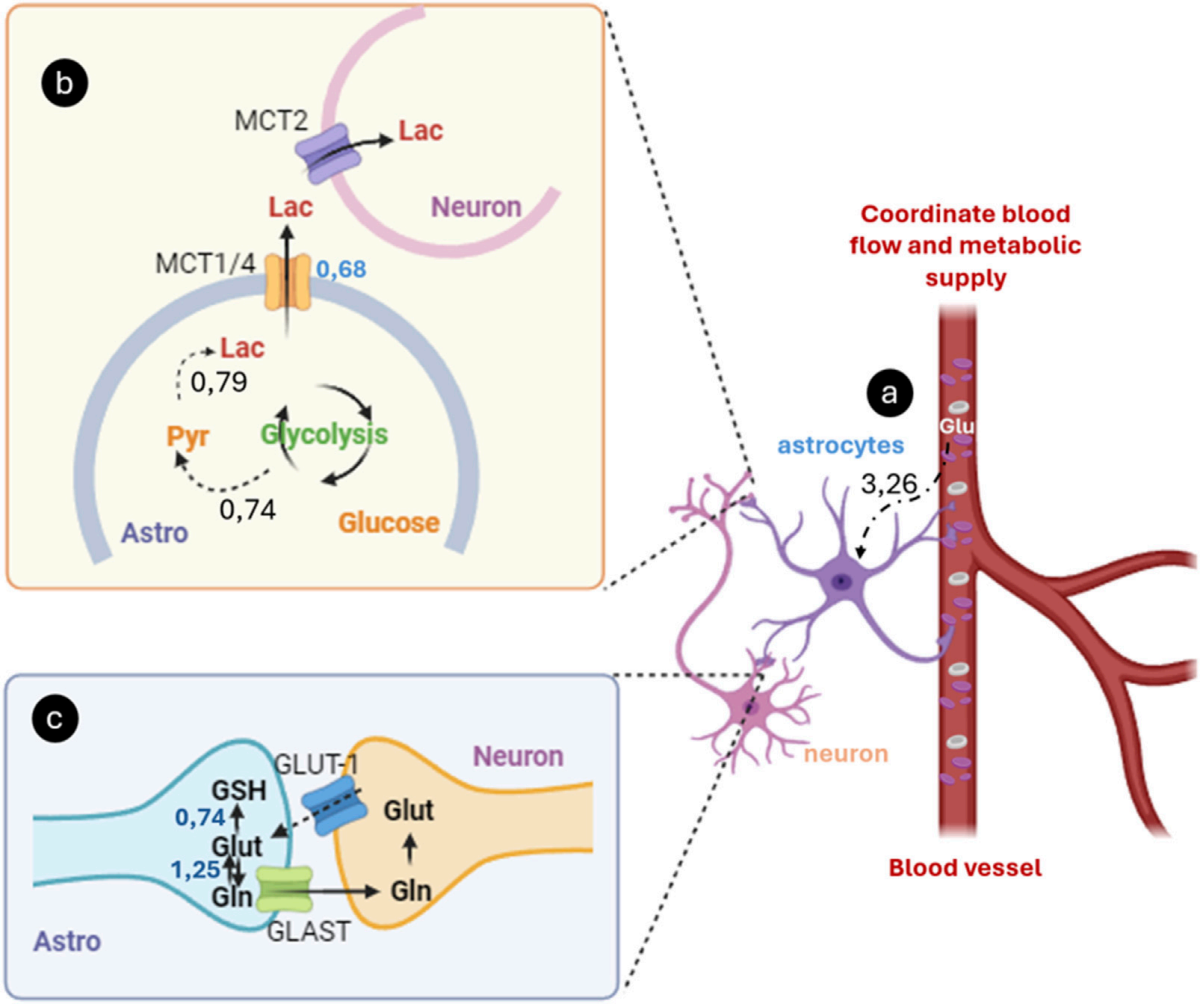
Astrocyte-Mediated glucose uptake, lactate production, and glutamate-glutamine cycling. **(A)** Astrocytes uptake glucose and other metabolites from the blood via specific transporters like GLUT. **(B)** Glucose is metabolizing it through glycolysis to produce lactate. This lactate is released into the extracellular space, where neurons absorb it as an energy source, particularly during periods of high synaptic activity; **(C)** Concurrently, astrocytes capture glutamate from the synaptic cleft using transporters, converting it into glutamine via glutamine synthetase. The non-excitotoxic glutamine is then shuttled back to neurons, where it is converted back into glutamate by glutaminase, enabling its reuse as a neurotransmitter. The numbers represent the material flow values (mmol gDW⁻^1^ h⁻^1^) reported by Mendoza model.

The fundamental role of astrocytes in the brain, which contrasts with the traditional neuron-centered view, highlights their importance in maintaining the metabolic and functional balance of the central nervous system (Mederos et al., 2018; Bélanger and Magistretti, 2009; Robertson, 2018). This perspective underscores the critical function of astrocytes in glucose uptake from blood vessels and its conversion into lactate, which is then used as a crucial energy source for neurons through oxidative phosphorylation (Souza et al., 2019) (Figure 6A). Since neurons have limited glycolytic capacity, they rely on astrocytic lactate to meet their energy needs (Beard et al., 2022). This mechanism is central to the astrocyte-neuron lactate shuttle hypothesis, in which astrocytes convert glucose into pyruvate through glycolysis (Marty-Lombardi et al., 2024; Alberini et al., 2019). This process can also involve the mobilization of stored glycogen (Figure 6B). Pyruvate is then converted into lactate by the enzyme lactate dehydrogenase and released into the extracellular space via monocarboxylate transporters (MCT1 and MCT4) (Alberini et al., 2019; Zhang et al., 2023; Jakoby et al., 2014; Henn et al., 2022). Neurons take up this lactate via MCT2, which is reconverted into pyruvate to enter the mitochondria and participate in oxidative phosphorylation, generating ATP (Zhang et al., 2023; Bélanger and Magistretti, 2009).

Lactate production in astrocytes is primarily driven by synaptic activity, particularly by releasing neurotransmitters like glutamate (Figure 6C) (Beard et al., 2022; Zhang et al., 2023). When glutamate is released at the synapse and taken up by astrocytes, it stimulates glycolysis (Henn et al., 2022). It increases lactate production, tightly linking this process to neuronal activity and changes in the extracellular microenvironment (Marty-Lombardi et al., 2024). Additionally, astrocytes facilitate the conversion of glutamate into glutamine, which is then transported back to neurons for recycling as an essential excitatory neurotransmitter (Rose et al., 2013; Schousboe et al., 2013; Pardo et al., 2013).

Our model emphasizes not only the role of astrocytes in lactate provision but also their critical involvement in the regulation of neurotransmission-related metabolites, specifically glutamate and glutamine cycling. Astrocytes actively take up synaptically released glutamate, preventing excitotoxicity, and convert it into glutamine (Sheng et al., 2013). This glutamine is then transported back to neurons for neurotransmitter recycling. In our model, the flux through these cycles is closely linked to synaptic activity and energy demands, demonstrating how astrocytes regulate neurotransmitter availability and balance in real time.

Our model also captures the regulation of critical neurotransmission-related metabolites, emphasizing the role of astrocytes in glutamate uptake and processing. Astrocytes uptake glutamate from the synaptic cleft via specific transporters, such as EAAT1 and EAAT2, to prevent its accumulation and excitotoxicity (Parkin et al., 2018). Once inside the astrocytes, glutamate is converted into glutamine by the enzyme glutamine synthetase (Hayashi, 2018). This conversion is crucial because it transforms glutamate, an excitotoxic neurotransmitter, into glutamine, which can be safely shuttled back to neurons.

The synthesized glutamine in astrocytes is exported to neurons via transporters such as SNAT3, where it is reconverted into glutamate by the enzyme glutaminase, allowing glutamate to be reused as a neurotransmitter in the synapse. This cycle, known as the glutamate-glutamine cycle, is essential for maintaining efficient excitatory neurotransmission and preventing excessive glutamate buildup in the synaptic cleft, which could lead to neuronal damage.

Quantitatively, the fluxes reported by the Mendoza model indicate that the transport of glutamine from astrocytes to neurons occurs at a rate of 1.25 mM gDW⁻^1^ h⁻^1^, while the conversion of glutamate into glutathione (GSH), a key antioxidant, occurs at a rate of 0.74 mM gDW⁻^1^ h⁻^1^ in astrocytes. This highlights a dual pathway for glutamate: one in which it is recycled in the glutamate-glutamine cycle (Bröer and Brookes, 2001), and another in which it is directed toward GSH synthesis in astrocytes, contributing to the regulation of the redox balance in the central nervous system (Bröer and Brookes, 2001; Zielińska et al., 2022).

As seen in Figure 6C, the model clearly represents how synaptic activity, particularly the release of glutamate, stimulates these metabolic processes. In addition to astrocytic lactate production, the representation of the glutamate-glutamine cycle and its role in neurotransmitter homeostasis and redox balance is also captured (Yamagata, 2022). The flux of metabolites between glutamate and GSH suggests that astrocytes play a critical role not only in supporting neuronal energy needs but also in providing antioxidant protection, thereby linking energy metabolism with the regulation of neurotransmission-related metabolites.

Although we lack experimental data on inflammatory flux levels observed in the model, it is possible to determine from our results that astrocytes can metabolize palmitic acid, a saturated fatty acid, through β-oxidation (Ng and Say, 2018). However, elevated levels of palmitic acid can induce pro-inflammatory responses in astrocytes, impairing autophagy, which may lead to cellular stress and contribute to neurodegenerative diseases (Vesga‐Jiménez et al., 2022; Ortiz-Rodriguez et al., 2019). Previous studies suggest that palmitic acid influences astrocyte lactate production, promoting increased generation while reducing glucose oxidation (Osorio et al., 2020; Oliveira et al., 2018; Bonvento and Bolaños, 2021). This shift toward greater reliance on glycolysis could represent an alternative energy generation pathway during metabolic stress.

The interaction between glucose and palmitic acid metabolism in astrocytes suggests that, while palmitic acid may not significantly alter glucose uptake, it can shift the metabolic balance in ways that could reduce lactate production rather than increase it, contrary to what might be expected (Gradisnik and Velnar, 2023; Morant-Ferrando et al., 2023). This reduction may stem from metabolic reprogramming under inflammatory conditions, where astrocytes may prioritize pathways other than glycolysis, such as fatty acid oxidation or responses to oxidative stress (Beard et al., 2022; Weightman Potter et al., 2019). Inflammatory signals triggered by palmitic acid can lead to mitochondrial dysfunction, impairing the efficiency of glucose oxidation and decreasing pyruvate availability for lactate production.

Although the effects of palmitic acid on lactate transport are not fully understood, evidence suggests that it may alter the activity of monocarboxylate transporters (MCTs) in astrocytes, potentially reducing the efficiency of lactate export to neurons (Angarita-Rodríguez et al., 2022; Escartin et al., 2007; Weightman Potter et al., 2019). Furthermore, palmitic acid’s effects on cell membrane fluidity could affect MCT activity and expression, especially in response to the oxidative stress it induces (Wang et al., 2012). This could further contribute to a reduction in lactate flux under inflamed conditions, as seen in our predictions.

Additionally, exposure to palmitic acid can increase oxidative stress, raising the demand for glutathione, an essential antioxidant (Morant-Ferrando et al., 2023; Wu et al., 2019; Nemecz et al., 2019). However, our findings indicate that under palmitic acid exposure, the demand for glutathione decreases, suggesting that other metabolic pathways might compensate for the oxidative stress or that astrocytes may reduce glutathione synthesis due to impaired metabolic functions. This shift could alter the availability of glutamine for other metabolic processes, as it is no longer being diverted significantly toward glutathione synthesis (Alnahdi et al., 2019). Maintaining a balance between these metabolites is critical for the proper functioning of astrocytes and their ability to support neurons through metabolic and antioxidant pathways (Morant-Ferrando et al., 2023). Disruption of this balance, as seen with excessive palmitic acid exposure, could negatively impact astrocyte function and brain health (Oliveira et al., 2018; Báez Castellanos et al., 2020).

Further studies are required to support our model’s predictions under inflammatory conditions. The inclusion of experimentally measured fluxes in astrocytes under inflammation would provide a robust comparison for validation, particularly in relation to palmitate metabolism and lactate production. The use of synthetic growth media, as opposed to fetal bovine serum (FBS), could also reduce variability in flux predictions and improve consistency across experiments. Moreover, integrating metabolomic data in future work could enhance our predictions by providing additional layers of information regarding metabolite availability and consumption (Morant-Ferrando et al., 2023). This approach could also facilitate the model’s adaptation to simulate other brain cell types, such as neurons or microglia, broadening its utility in studying neurodegeneration.

Adopting a holistic perspective, our model emphasizes the importance of the glutamate-glutamine cycle in the brain’s metabolic balance. It highlights glutamine’s role not only as a neurotransmitter precursor but also as an energy intermediate through its conversion into α-ketoglutarate in the Krebs cycle (Shen et al., 2009). This approach reaffirms the relevance of astrocytes in regulating neurotransmission and energy homeostasis in the central nervous system.

Our results demonstrated that our model achieved a prediction accuracy of 96.88% compared to the previous model published by Osorio et al. (2020), which integrated transcriptomic and proteomic data and achieved an accuracy of 84.38% relative to the experimentally predicted value by Prah et al. (2019). This accuracy was calculated by determining the ratio of the difference between the model’s predicted values and the experimental data, reflecting the model’s improved ability to predict metabolic fluxes in this specific context.

However, the use of a universal biochemical reaction database for gap filling, instead of Recon3D, may have introduced bias. We acknowledge this potential limitation and suggest that using Recon3D exclusively in future work may help address this concern by providing reactions that are better suited for astrocytic metabolism.

The use of techniques such as PCA to combine transcriptomic and proteomic data in the reconstruction of specific GEMs has shown promising results, especially compared to previous methods like iMAT and exp2flux (Angarita-Rodríguez et al., 2022, Angarita-Rodríguez et al., 2024). These advances in GEM contextualization underscore the utility of integrating multiple data layers to create more accurate models applicable to studying complex pathologies such as neurodegenerative diseases. The human astrocyte GEM we have developed is, to date, the most complete in terms of the number of genes, proteins, reactions, and metabolites, and its validation with experimental data reinforces its usefulness as a tool to explore new hypotheses regarding astrocyte function in health and disease. Nevertheless, the advantages of one approach over the other still require further investigation, as network reduction may be necessary in cases where metabolic pathways need modifications without experimental data support.

While the integration of transcriptomic and proteomic data has improved predictions in our model, it is important to acknowledge the limitations of relying solely on these data sources. The use of ribosomal profiling as an additional method to bridge the gap between the transcriptome and proteome has shown promising results (Ebrahim et al., 2016). However, ribosomal profiling data is not widely available, which can affect the robustness of our findings. Current approaches often prioritize one data type over the other, potentially leading to inaccuracies in model predictions. Future research should explore the application of ribosomal profiling across various cell types, including astrocytes and neurons, to provide a more precise measurement of translation and enhance the integration of multi-omics data in genome-scale metabolic models (GEMs). This approach could deepen our understanding of cellular metabolism and improve the accuracy of metabolic models.

Furthermore, while the integration of multiple data layers has led to improved predictions, the quality of the input data remains a key factor in determining the accuracy of the model’s outcomes. Although we lack experimental flux values to fully validate the model’s behavior under inflammatory conditions, the model still offers higher resolution estimates of fluxes. Its flexible structure allows easy adaptation to neuronal metabolic networks or even co-culture systems, enabling the separation of distinct cellular compartments. Expanding the model to describe metabolic interactions between astrocytes and neurons would involve a significantly larger number of reactions and unknown fluxes, which would necessitate a more comprehensive experimental dataset, particularly measurements of specific metabolic rates and reserves for astrocytes and neurons independently.

One potential source of error in our predictions is the variability in the nutritional medium, such as FBS, whose composition can fluctuate (Yang and Xiong, 2012). This variability could explain discrepancies between model predictions and experimental data. Therefore, we recommend measuring the concentrations in the nutritional medium and adjusting the model, accordingly, preferably using a synthetic growth medium with a fixed composition.

Finally, Recon3D, as the base GEM, allowed us to reconstruct the most comprehensive human astrocyte GEM to date regarding the number of genes, proteins, reactions, and metabolites. Its contextualization with multi-omics data has improved its predictive performance, as evidenced by comparing our results with state-of-the-art astrocyte GEMs.

Future research should explore the integration of additional omics data, such as metabolomics, to further enhance the predictive capabilities of GEMs. Expanding the model to include a broader array of omics data will likely improve the accuracy of predictions and provide deeper insights into the metabolic interactions between astrocytes and neurons. Additionally, future studies should examine the broader implications of these findings, particularly in the context of metabolic diseases and neurodegenerative disorders. This holistic approach could lead to the discovery of novel therapeutic targets and a better understanding of the metabolic underpinnings of brain health.

Our current methodology, while focused on astrocytes, holds potential for expansion to model other brain cell types, such as neurons and microglia, which would enable a more comprehensive view of brain metabolism in health and disease. Additionally, developing a neuron-astrocyte interaction model using this approach could provide insights into the cooperative metabolic processes critical for maintaining homeostasis in the central nervous system. Future work could leverage the current framework to explore metabolic crosstalk, particularly under pathological conditions such as neurodegenerative diseases. The integration of interaction data, combined with multi-omics inputs, would facilitate a holistic perspective on how cellular interactions influence brain metabolism and function.

This GEM will be a crucial tool for studying the metabolic functions of astrocytes and their relationship with neurodegeneration. By integrating and contextualizing multi-omics data, the model can help generate new hypotheses regarding both pathological and normal processes in these cells. As research progresses, this model may also guide the exploration of therapeutic interventions and contribute to the development of strategies aimed at preserving or restoring brain health.

## Data Availability

The datasets presented in this study can be found in online repositories. The names of the repository/repositories and accession number(s) can be found in the article/Supplementary Material.
