# Supplementary material for: Improvement in the prediction power of an astrocyte genome-scale metabolic model using multi-omic data
Source: Front Syst Biol. 2025 Jan 3;4:1500710. doi: 10.3389/fsysb.2024.1500710 (PMC12341997; doi:10.3389/fsysb.2024.1500710)
Supplement: Supplementary file 1 [file Supplementaryfile1.docx]

Supplementary Material

## Tables

## Supplementary Tables

# Supplementary Table 1. Nutritional input composition. Composition and concentration of the metabolites defined as the nutritional input of the GEM (Branzoi et al., 2010; Yang and Xiong, 2012; Tejera et al., 2020).

| **Metabolite** | **Concentration** | **Metabolite** | **Concentration** |
| --- | --- | --- | --- |
| Glycine | -0.03 mM | L-Arginine | -846.7 µM |
| L-Glutamine | -584 mM | L-Valine | -451.3 µM |
| L-Isoleucine | -105 mM | L-Isoleucine | -415.2 µM |
| L-Leucine | -105 mM | L-Leucine | -450.1 µM |
| L-Methionine | -0.03 mM | L-Lysine | -624.1 µM |
| L-Phenylalanine | -66 mM | L-Proline | -149.9 µM |
| L-Serine | -42 mM | L-Threonine | -448.8 µM |
| L-Threonine | -95 mM | L-Cystine | -130.2 µM |
| L-Valine | -94 mM | L-Glutamine | -2.5 mM |
| 7,8-Dihydrofolate | -4 mM | Hypoxanthine | -15.4 µM |
| L-Alanine | -50 µM | Lipoate | -508.9 µM |
| Myo-Inositol | -72 mM | Triglyceride | -1.26 mM |
| D-Glucose | -4.5 mM | Phosphatidylserine | -0.72 mM |
| Myo-Inositol | -69.9 µM | Cholesterol | -1.1 mM |
| Thymidine | -1.5 µM | Oxygen | -10 mM |
| Riboflavin | -581.9 nM | Nitric Oxide | -10 mM |
| Pyridoxal | -150.7 nM | Cytidine | -1.3 µM |
| L-Cysteine | -100.2 µM | Uridine | -5.1 µM |
| L-Asparagine | -56.8 µM | Urate | -130 µM |
| L-Tryptophan | -44.2 µM | Hypoxanthine | -74.7 µM |
| Nicotinamide Adenine Dinucleotide | -4 mM | Homocitrulline | -30.1 µM |

**Figures**

##### Supplementary Figures

**B)**

**A)**


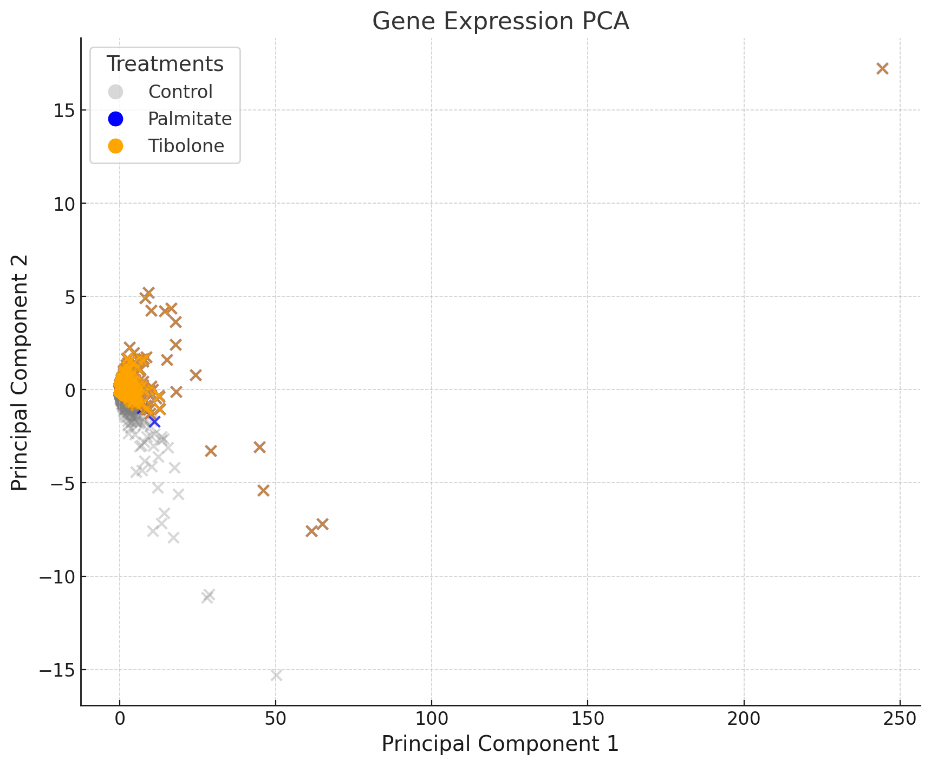

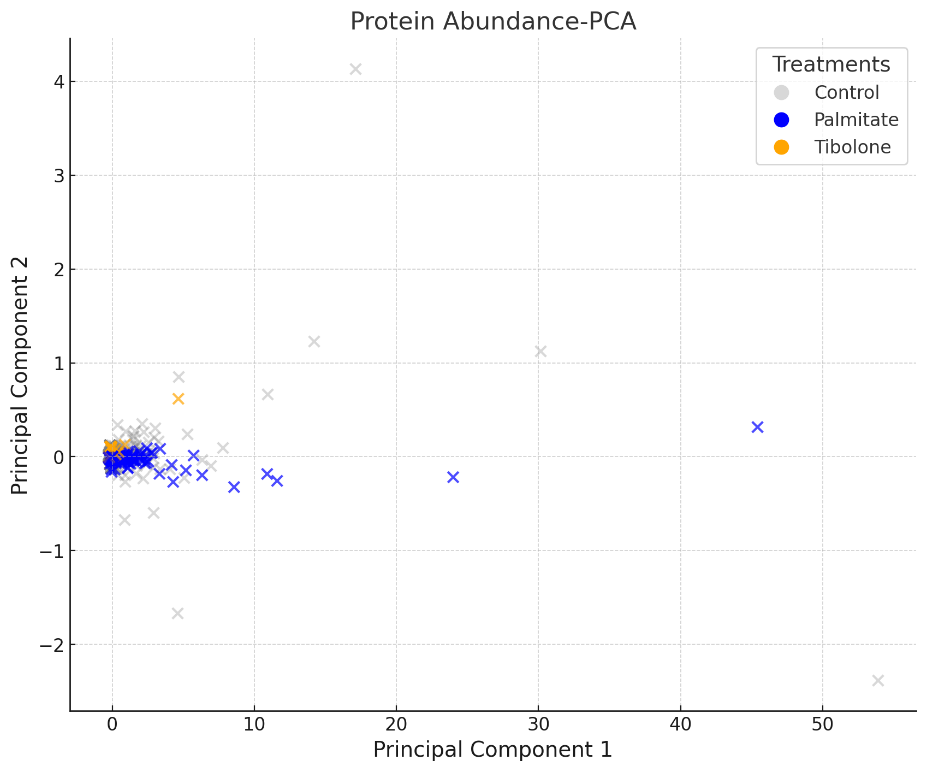


**Supplementary Figure 1. A-B) Principal Component Analysis (PCA) of Gene Expression and Protein Abundance Data:** *Visualization of variance and clustering patterns across experimental conditions (Palmitate, Tibolone, and Control) prior to data integration. In Gene expression PC1 explains 97.97% of the variance; PC2 explains 1.99% of the variance; In protein Abundance PC1 explains 97.03% of the variance; PC2 explains 1.57% of the variance.*
